# Ladder-based resistance training with the progression of training load altered the tibial nerve ultrastructure and muscle fiber area without altering the morphology of the postsynaptic compartment

**DOI:** 10.3389/fphys.2024.1371839

**Published:** 2024-04-17

**Authors:** Walter Krause Neto, Wellington Silva, Tony Oliveira, Alan Vilas Boas, Adriano Ciena, Érico Chagas Caperuto, Eliane Florencio Gama

**Affiliations:** ^1^ Department of Morphology and Genetics, Universidade Federal de São Paulo, São Paulo, Brazil; ^2^ Depatment of Physical Education, Laboratory of Human Movement, Universidade São Judas Tadeu, São Paulo, Brazil; ^3^ Department of Physical Education, Laboratory of Morphology and Physical Activity, Universidade Estadual Paulista Júlio de Mesquita Filho, São Paulo, Brazil

**Keywords:** exercise, tibial nerve, motor unit, neuromuscular junction, skeletal muscles

## Abstract

Scientific evidence regarding the effect of different ladder-based resistance training (LRT) protocols on the morphology of the neuromuscular system is scarce. Therefore, the present study aimed to compare the morphological response induced by different LRT protocols in the ultrastructure of the tibial nerve and morphology of the motor endplate and muscle fibers of the soleus and plantaris muscles of young adult Wistar rats. Rats were divided into groups: sedentary control (control, n = 9), a predetermined number of climbs and progressive submaximal intensity (fixed, n = 9), high-intensity and high-volume pyramidal system with a predetermined number of climbs (Pyramid, n = 9) and lrt with a high-intensity pyramidal system to exhaustion (failure, n = 9). myelinated fibers and myelin sheath thickness were statistically larger in pyramid, fixed, and failure. myelinated axons were statistically larger in pyramid than in control. schwann cell nuclei were statistically larger in pyramid, fixed, and failure. microtubules and neurofilaments were greater in pyramid than in control. morphological analysis of the postsynaptic component of the plantar and soleus muscles did not indicate any significant difference. for plantaris, the type i myofibers were statistically larger in the pyramid and fixed compared to control. the pyramid, fixed, and failure groups for type ii myofibers had larger csa than control. for soleus, the type i myofibers were statistically larger in the pyramid than in control. pyramid and fixed had larger csa for type ii myofibers than control and failure. the pyramid and fixed groups showed greater mass progression delta than the failure. We concluded that the LRT protocols with greater volume and progression of accumulated mass elicit more significant changes in the ultrastructure of the tibial nerve and muscle hypertrophy without endplate changes.

## Introduction

The peripheral nervous system (PNS) is composed of nerves and nerve ganglia, whose functions can be summarized as transmitting motor commands from the central nervous system (CNS) to the skeletal muscles (Pikatza-Menoio et al., 2021), transmitting sensory information via the afferent pathway to the CNS (Santuz and Akay, 2023); and monitor and adjust autonomic functions (Nelemans and Dogterom, 1954; Goldstein, 2001). Functionally, all CNS and PNS communication is conducted by electrochemical signals, such as action and graded potentials (Stuart et al., 1997; Sengupta et al., 2014; Tavee, 2019; Metcalfe et al., 2021).

The PNS sends and receives information from skeletal muscles through highly organized structures called peripheral nerves (Crockett and Edgerton, 1975; Ito et al., 2014). Each peripheral nerve is a cable-like anatomical structure formed by multiple specialized nerve fibers, called axons, whose function is to conduct electrical impulses and enable the axoplasmic transport of molecules for homeostatic purposes (Rigoard et al., 2009; Debanne et al., 2011).

Morphologically, peripheral nerves contain myelinated and unmyelinated axons with different sizes and functions (Debanne et al., 2011; Feltri et al., 2016). Each myelinated axon is surrounded by multiple folds of myelin (a lipid membrane rich in glycophospholipids and cholesterol) in a spiral shape, isolating the cell membrane from the axon and allowing rapid electrical transmission. The myelin sheath is produced by Schwann cells (SC), responsible for insulating and supplying nutrients to the axons. In addition, peripheral nerves also contain small clusters of unmyelinated afferent fibers (class C fibers) (Craig, 2002; Murinson and Griffin, 2004). In the intracellular compartment, axons are formed by microtubules and neurofilaments, providing transport routes for important molecules between the cell body and its nerve endings (Baas, 2000; Baas, 2002; Fenn et al., 2018).

Upon reaching the skeletal muscle, the nerve endings form anatomically and physiologically active structures called the neuromuscular junction (NMJ). The NMJ is a synaptic connection site between the terminal end of the motor neuron and the muscle fiber. The structure of the NMJ can be subdivided into three compartments: the presynaptic neuron, the synaptic cleft, and the motor endplate (Wilson and Deschenes, 2005).

The myelinated motoneuron forms a complex of branched nerve endings (between 100 and 200) called synaptic buttons at the nerve terminal. In the nerve terminal membrane, there are areas of membrane thickening called active zones, whose synaptic vesicles contain the neurotransmitter acetylcholine (ACh). Upon the arrival of an action potential, voltage-gated calcium channels open, and a series of events cause exocytosis of ACh from synaptic vesicles into the synaptic cleft (Slater, 2017; Gorzi et al., 2021). After its release, ACh interacts with nicotinic receptors on the motor endplate, propagating the action potential throughout the sarcolemma.

Morphologically, the motor endplate is the thick portion of the sarcolemma plasma membrane, whose depressions form the so-called junctional folds. Interestingly, the nerve endings do not penetrate the motor endplate despite fitting into the junctional folds. Instead, the junctional folds have concentrated nicotinic ACh receptors, whose connections lead to the opening of voltage-gated ion channels, propagating electrical energy throughout the muscle fiber (Wilson and Deschenes, 2005).

Recent work has suggested that the increase or absence of stimuli can elicit changes in the neuromuscular system (Wilson and Deschenes, 2005; Krause Neto et al., 2017; Deschenes et al., 2021; Krause Neto et al., 2021; Deschenes and Stock, 2022; Krause Neto et. 2015). Furthermore, stimuli with different intensities and training volumes cause different magnitudes of response in the muscular strength of healthy individuals (Lixandrão et al., 2018). This suggests that different training protocols might induce different physiological adjustments in the neuromuscular system and possibly different morphological responses.

Regular resistance training (RT) causes, in most cases, an increase in strength, power, and muscle mass. According to Scarpelli et al. (2022) and Nóbrega et al. (2023), the magnitude of muscle hypertrophy in human beings seems to be determined by adequate monitoring of the progression of the training load. Also, RT strategies until voluntary muscle failure, whether with traditional protocols or advanced training methods, significantly induce similar hypertrophic responses when accumulated mass progression is equalized (Angleri et al., 2017). Yet, Damas et al. (2019) and Schoenfeld et al. (2015) demonstrated that RT protocols trained to exhaustion, even with different training volumes and intensity, tend to hypertrophy similarly. On the other hand, high-intensity resistance training induces greater strength gains than low-intensity training, even when accumulated mass is equalized (Schoenfeld et al., 2017). This evidence demonstrates that RT protocols with high effort and adequate progression of training load are fundamental for morphological changes in skeletal muscle; however, it might induce different morphological responses in other neuromuscular compartments.

At an experimental level, ladder-based resistance training (LRT) induces muscle hypertrophy like human RT (Lourenço et al., 2020). According to Krause Neto et al. (2022a), the progression of the external training load, either by total or relative training mass, is also critical for rodent muscular hypertrophy. However, the effect of RT in other neuromuscular sites, such as NMJ morphology, of young models remains controversial and could not be explained by muscle fiber training-induced hypertrophy (Deschenes et al., 2000; Deschenes et al., 2015). Although the effects of resistance training on muscular hypertrophy are well documented in the literature, little is known about the adjustments in the morphology of the peripheral nerve and motor endplate of different muscles subjected to other resistance training protocols.

Resistance training induces significant neurophysiological adaptations, a potential neuroplasticity stimulator (Hortobágyi et al., 2021; Škarabot et al., 2021). At the PNS level, LRT with higher training volume or intensity could lead to more significant changes in the ultrastructure of the peripheral nerves (Carbone et al., 2017; Krause Neto et al., 2022b). However, although the neuroplasticity of the CNS induced by training, especially running training, is demonstrated in the literature (Nowacka-Chmielewska et al., 2022), it is not known to what extent RT, specifically from different training protocols, causes neuroplasticity in the PNS different sites, such as peripheral nerve and endplate morphology. The results of this study may guide training and healthcare professionals, such as sports professionals and rehabilitation centers, to choose training protocols that are more appropriate and specific to the desired outcomes.

Therefore, this study had the following hypotheses: a) all groups, regardless of the characteristics of the training protocol, will present significant changes in the general morphology of the tibial nerve, and b) the LRT groups with greater progression of accumulated mass will demonstrate significant changes in the morphology of motor end-plate and muscle hypertrophy.

Therefore, this study aimed to compare the morphological adjustments induced by different LRT protocols in the tibial nerve, motor endplate, and muscle fibers of the soleus and plantaris muscles of young adult Wistar rats.

## Methods

The ethics committee on using animals for research at Universidade São Judas Tadeu authorized this experiment under protocol 022/2016.

Thirty-six rats were divided into groups: sedentary control (CONTROL, n = 9), resistance training with a predetermined number of climbs and progressive submaximal intensity (FIXED, n = 9), resistance training with a high-intensity pyramidal system with a predetermined number of climbs (PYRAMID, n = 9) and resistance training with a high-intensity pyramidal system to failure (FAILURE, n = 9).

During the experiment, four-month-old male Wistar rats were housed in polypropylene boxes (up to five rats) under controlled lighting conditions (22 °C—a light-dark cycle of 12 h). All rodents received commercial rodent chow (Nuvilab CR-1 [22% protein]) and water *ad libitum*. However, the amount of food and water ingested by each rodent was not controlled.

### Ladder-based resistance training protocol

The rodents were housed and trained in an inverted light-dark cycle environment.

**FIGURE 1:**
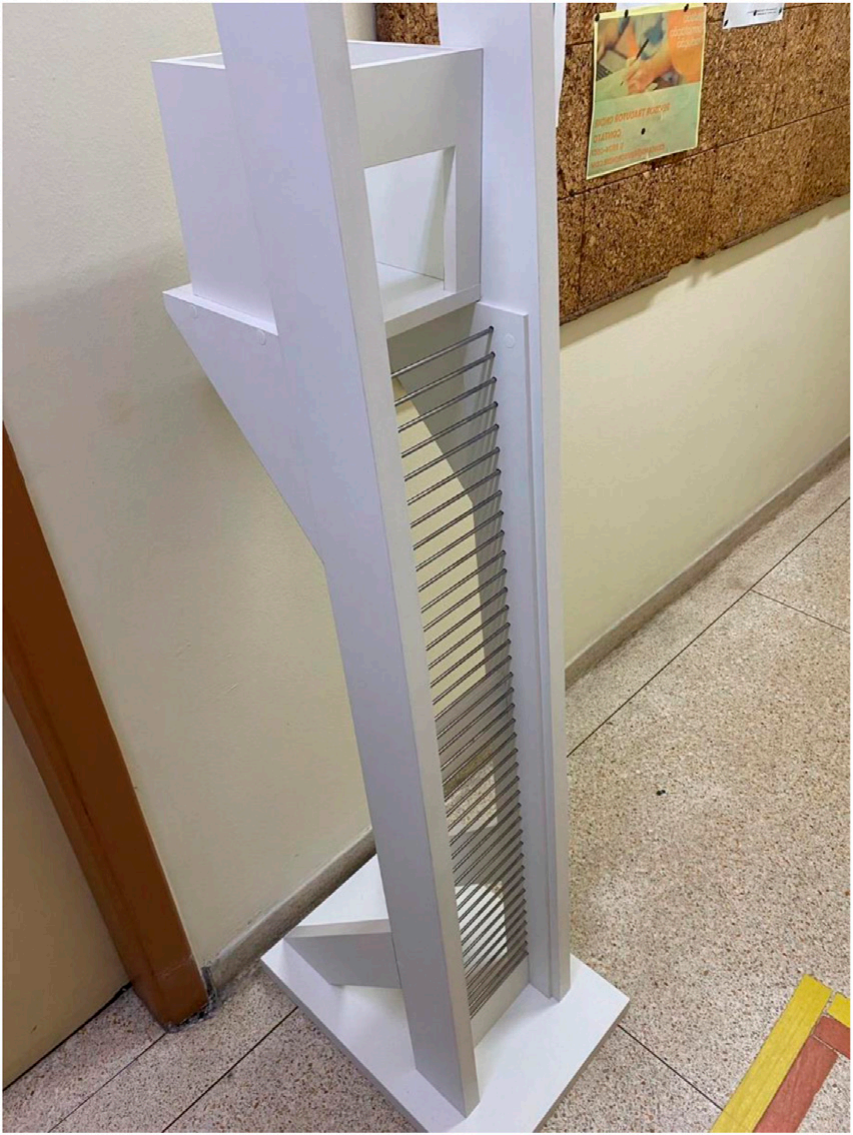
Illustration of the ladder-based training equipment.

In the first week, the rodents climbed the ladder (Figure 1) (wooden, with iron steps, 110 cm high, 80° inclination, and 2 cm of distance between each step) without attached weight for five consecutive days, three times per session, starting from three different positions (upper, middle, and lower third).

All rodents were submitted to the maximal progressive climbing test in the second week. First, the rodents climbed the ladder without any additional weight. Then, 10% of the weight relative to the rodent’s body mass (B.m) was added to the rodent’s tail at each successful climb, with 2-min intervals between each climb, until the rodent reached voluntary failure (the rodent’s inability to climb the ladder for two consecutive attempts with the same tied weight). If the rodent stopped climbing the ladder, a light tap on its tail was given as a stimulus to continue climbing to the top. However, if the rodent refused to climb, even after touching its tail, a 2-min interval was given, and a new attempt at climbing was allowed. In case of a further refusal, the training session was interrupted.

Furthermore, it is necessary to clarify that no rodent received incentive or motivation (food/water) to climb the ladder. Therefore, the maximum mass (MM) carried to the top of the ladder on the last successful attempt was considered the maximum load (MM-PRE). After eight training weeks, the rodents were again submitted to the maximal progressive climbing test (MM-POST) 3 days after the last LRT session.

The FIXED group underwent the LRT protocol of moderate volume and progression of training intensity suggested by the American College of Sports Medicine (ACSM, 2009). First, the rodents climbed the ladder six times with an intensity of 60 %MM. For subsequent sessions, if the rodent completed the number of six climbs, the weight tied to each rodent’s tail was adjusted between 2% and 5%. A 2-min break was used between each successful climb.

The PYRAMID group climbed the ladder six times, with progressive increases in intensity after every two successful climbs, as Harris et al. (2010) suggested. Therefore, each rodent climbed the ladder with 2 × 50%MM, 2 × 75%MM, and 2 × 100%MM. From there, the rodents climbed the ladder twice with 100%MM +30 g of weight tied to their tail, totaling eight successful climbs. We recalculate the following training session percentages from this new maximum loaded weight. However, if the rodent did not complete the eight climbs, we kept the weights for the next session. The rodents were given a 2-min break between each climb.

The FAILURE group was submitted to the resistance training protocol that Hornberger and Farrar (2004) suggested. Thus, the rodents climbed the ladder using a pyramidal training model, with intensities equivalent to 50%MM, 75%MM, 90%MM, and 100%MM. From 100%MM, we add 30 g of extra weight to the rodent’s tail on every successful climb. The training session was conducted until the rodent was voluntarily unable to climb the ladder for two consecutive attempts (failure). Between each climb, the rodent had 2 min of rest.

All groups trained a week thrice, on non-consecutive days, for 8 weeks.

### Preparation of material for analysis

The closed chamber CO_2_ inhalation euthanasia method was used in this experiment, as described in Krause Neto et al. (2022). After euthanasia, an incision from the back of the rodent’s right knee to its ankle was made to expose the tibial nerve, the plantaris (PL), and the soleus (SL) muscle. We chose the tibial nerve because it innervates rodents’ predominant muscles in the posterior region of the lower limbs, including PL and SL. In addition, the PL and SL muscles were selected to respond to the LRT stimulus (Lourenço et al., 2020).

### Transmission electronic microscopy

A 0.5 cm fragment of the tibial nerve was removed after euthanasia. Then, each segment was fixed with 2.5% glutaraldehyde in phosphate buffer (0.2M, pH 7.3) for 3 hours. Next, the tissue was washed three times with the same buffer solution for 5 minutes. Then, we placed the tissue in a 1% osmium tetroxide solution in phosphate buffer for 2 hours. Overnight, the tissue remained in 0.5% uranyl acetate. In the morning, the tissue was washed with a buffer substance and dehydrated in an increasing series of alcohol and propylene oxide for 8 hours under rotation. Next, the tissue was placed in pure resin (Spurr), and the nerve fibers were transversely sectioned. In this step, we let the material rest for 5 hours. Next, the tissue remained in the same resin at 60°C for 3 days. Finally, semi-thin sections were made to prepare histological slides stained with toluidine blue. After selecting the fields for analysis, ultrathin cuts were created with a diamond knife in an ultramicrotome (Sorvall MT-2) and contrasted with uranyl acetate and lead citrate. The final material was photographed in an electronic transmission microscope (Morgagni 268D).

All outcomes analyzed in this study followed standardized criteria (Carbone et al., 2017; Krause Neto et al., 2017; Bessaguet et al., 2018; Krause Neto et al., 2022b). We took fifteen microphotographs per nerve per rodent in each group according to the protocol by Krause Neto et al. (2022b). All images were photographed following a cross pattern, from top to bottom and left to right (150 myelinated and unmyelinated fibers per rodent).

**FIGURE 2:**
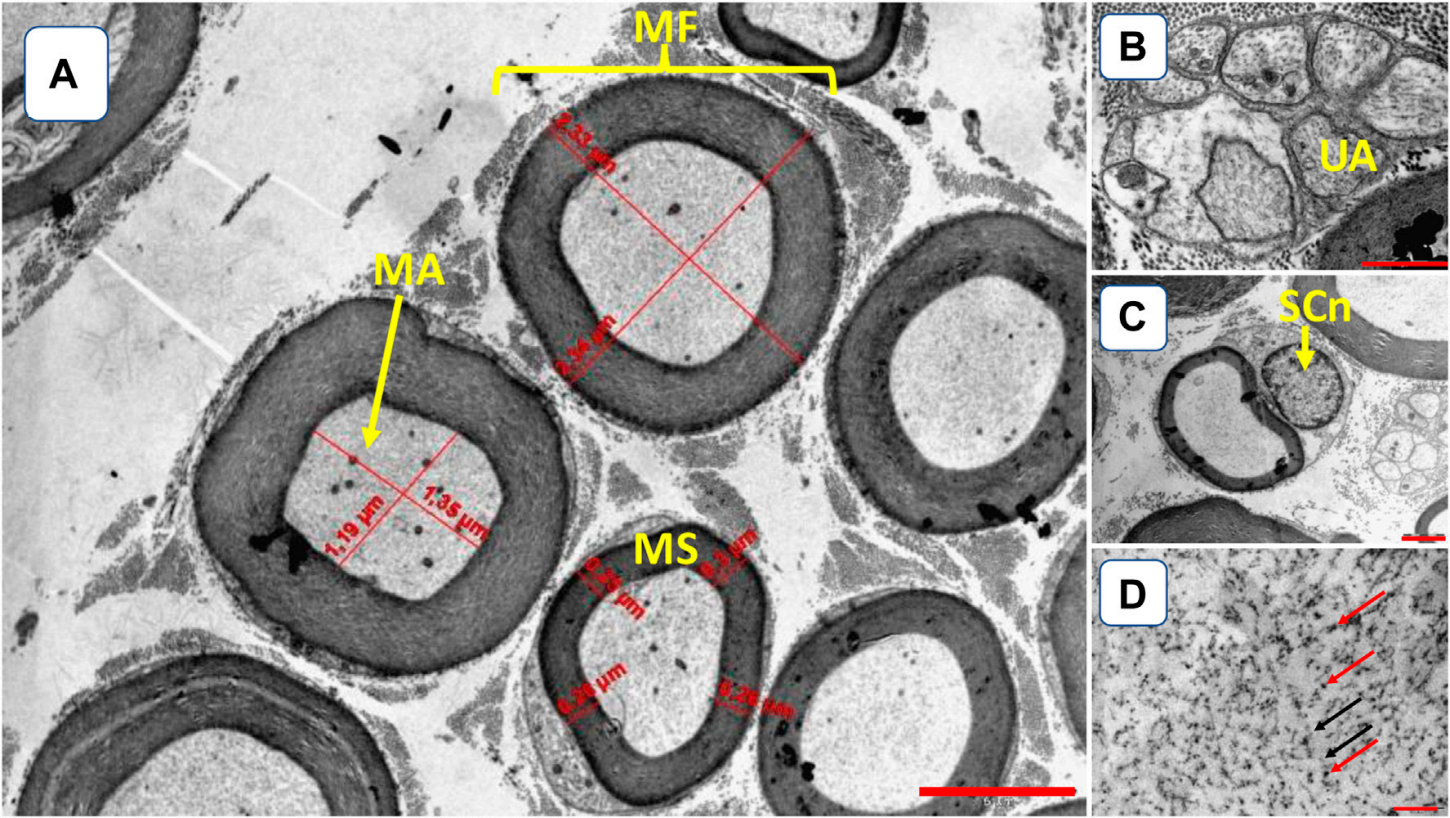
Illustration of tibial nerve morphology. **(A)** myelinated fibers (MF); myelinated axons (MA); myelin sheath (MS) [3,000x amplification, bar 5 µm]; **(B)** UA–unmyelinated axon (UA) [12000x amplification, bar 2 µm]; **(C)** Schwann cell nuclei (SCn, 10000x amplification, bar 2 µm); **(D)** microtubules (red arrows) and neurofilaments (black arrows) [22000x amplification, bar 0.025 µm].

We quantified the following parameters (Figure 2): the diameter of myelinated axons and fibers (µm), the diameter of unmyelinated axons (µm), the diameter of Schwann cell nuclei (µm), and the thickness of the myelin sheath (µm). We averaged the largest and smallest diameters to measure myelinated axons and fibers (3,000x magnification), unmyelinated axons (×12000 magnification), and SC nuclei (between 5 and 10 nuclei per rodent and at ×10000 magnification). We used numerical density to count neurofilaments and microtubules (×22000 magnification). The thickness of the myelin sheath (3,000x magnification) was calculated from the difference between the diameter of the axon and the myelinated fiber. We measured these outcomes using the Axiovision 4.8 program (Carl Zeiss, Germany).

### Cytofluorescence

We used a fluorescence microscope (Olympus BX61) from the Laboratory of Bacterial Genetics (UNESP-Rio Claro). Soon after extraction and cleaning, we removed the middle third of the PL and SL muscle, cryo-fixing it in liquid nitrogen and keeping it in a freezer tank at −80°C. To prepare the material, we made longitudinal cuts of 100 µm thickness using a cryotome at a temperature of −20°C. First, slides were pretreated in a 3% ethylenediaminetetraacetic acid solution to prevent tissue contraction. Next, the tissue was washed (6 × 5 min) in phosphate-buffered saline (PBS) containing 1% bovine serum albumin (BSA). During the night, the cuts were incubated for 16 hours (humidified chamber at 4 °C) in a solution with rhodamine-conjugated α-bungarotoxin (BTX; Molecular Probes, Eugene, OR - T-1175), diluted 1:600 PBS. Next, the material was finally washed (6 × 5 min) before being coated with Pro long (Molecular Probes, Eugene, OR - P10144), and the coverslip was applied.

**FIGURE 3:**
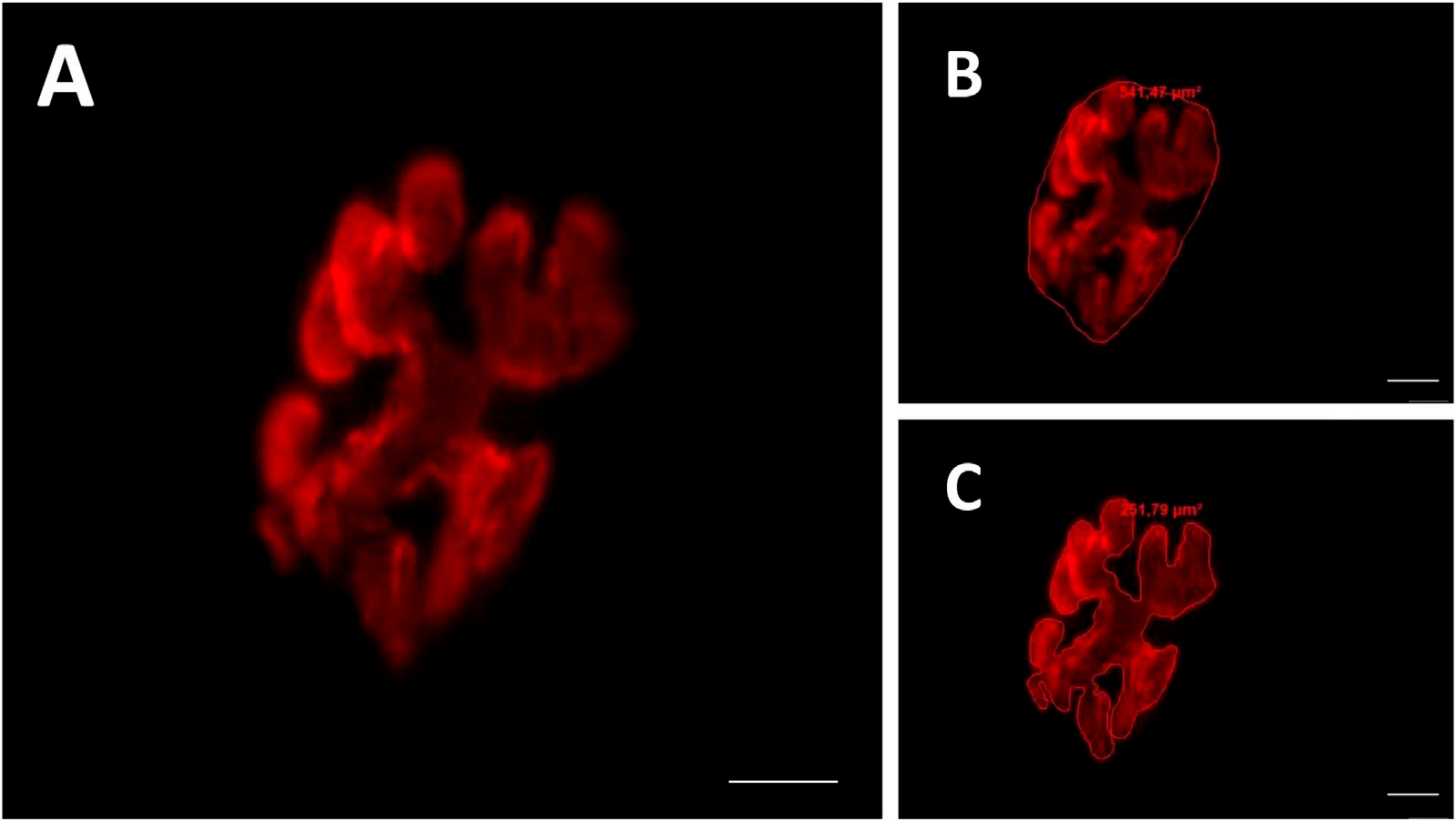
Representative image of the postsynaptic compartment (motor endplate). Red labeling of acetylcholine (ACh) receptors with α-bungarotoxin conjugated rhodamine **(A)**. Manually drawn lines for determination of the total perimeter, or the length covering the entire endplate composed of groups of stained receptors and non-stained regions interspersed within these groups **(B)**, stained perimeter, or composite length, which includes the recipients stained with uncorrected regions interspersed between groups of recipients, the stained area, or the areas occupied by cumulative groups of recipients Ach **(C)**. Increase by 800x.

We captured between fifteen and twenty photos of each muscle containing an endplate, with a magnification of ×800 (Figure 3). In the postsynaptic component, we measured the total perimeter (µm) [length encompassing the entire endplate composed of groups of stained receptors and unstained regions], stained perimeter (µm) [length composed of traces around groups of labeled receptors], total area (µm^2^) [receptors stained together with unstained regions], stained area (µm^2^) [areas occupied by cumulative groups of ACh receptors] and endplate dispersion [value calculated from dividing the stained area of the end plate by its total area and multiplied by 100] (Deschenes et al., 2013). For this analysis, we used the Axiovision 4.8 software.

### Histochemistry

To prepare histochemical slides, we collected, cleaned, weighed, and cryofixed in liquid nitrogen samples from the belly of the PL and SL muscles. The material remained at −80 °C until analysis.

The sample from each muscle was sectioned into cross-sections (10 µm thick; Cryostat HM 505 E, MICROM™). To prepare the material at pH 4.6, the tissue was pre-incubated in a buffered solution of 0.1 M sodium acetate and 10 mM ethylenediaminetetraacetic acid (EDTA) for 10 min at 4 °C. Then, the material was incubated in a solution containing ATP (10 mg), glycine/NaCl buffer (10 mL), CaCl^2^ (pH 9.4), and dithiothreitol for 30 min. Next, the material was washed in distilled water, incubated for 7 min in 2% cobalt chloride, washed in distilled water, and dehydrated in a series of alcoholic concentrations, ending with xylene (Cornachione et al., 2008).

We analyzed microphotographs of each group’s PL and SL muscles (n = 80–100/fiber type/rat/group) at a final magnification of ×400. The fiber cross-sectional area (fCSA) of types I and II was quantified using the Axiovision software version 4.8 coupled to a light microscope (Zeiss).

### Statistical analysis

Data are presented as mean and standard deviation. To analyze the normality of the data, we used the Shapiro-Wilk test. To compare the ultrastructural, cytofluorescence, histochemistry, and training data between groups, we used one-way ANOVA (Tukey *post hoc*). We used SPSS version 21.0; the significance level was *p* ≤ 0.05.

## Results

### Tibial nerve morphology

Table 1 presents data from tibial nerve morphology. The statistical results of the analysis of the ultrastructure of the tibial nerve indicate that resistance training significantly altered myelinated fibers diameter (F = 5.912, *p* = 0.002), myelinated axon diameter (F = 3.178, *p* = 0.037), myelin sheath thickness (F = 9.400, *p* < 0.001), Schwann cells nuclei diameter (F = 7.914, *p* < 0.001), and neurofilaments (F = 4.956, *p* = 0.006).

**TABLE 1:** Ultrastructural characteristics of the tibial nerve of the sedentary control (CONTROL), a predetermined number of climbs and progressive submaximal intensity (fixed), a high-intensity pyramidal system with a predetermined number of climbs (pyramid), and high-intensity pyramidal system to failure (failure) groups.

Variable	Control	Fixed	Pyramid	Failure
Myelinated fibers diameter (µm)	2.17 ± 0.6	2.81 ± 0.33^a^	2.93 ± 0.36^a^	2.74 ± 0.34^a^
Myelinated axons diameter (µm)	1.67 ± 0.54	2.08 ± 0.35	2.21 ± 0.3^a^	2.07 ± 0.38
Unmyelinated axons diameter (µm)	0.17 ± 0.03	0.18 ± 0.03	0.18 ± 0.03	0.19 ± 0.04
Myelin sheath thickness (µm)	0.5 ± 0.12	0.72 ± 0.1^a^	0.73 ± 0.11^a^	0.67 ± 0.09^a^
Schwann cells nuclei diameter (µm)	0.48 ± 0.13	0.69 ± 0.11^a^	0.72 ± 0.1^a^	0.66 ± 0.11^a^
Schwann cells nuclei (n°)	1.4 ± 0.74	1.71 ± 0.79	1.78 ± 0.9	1.89 ± 1.49
Microtubules (n°)	393.11 ± 61.31	468.56 ± 93.18	520.33 ± 121.34^a^	455.34 ± 89.04
Neurofilaments (n°)	1261.89 ± 225.13	1463.78 ± 230.85	1697.11 ± 275.01^a^	1393.44 ± 248.98

Data are presented as mean and standard deviation—legend.

^a^ Statistically different than control.

Myelinated fibers were statistically larger in pyramid (*p* = 0.003), fixed (*p* = 0.014), and failure (*p* = 0.031) groups compared to control.

Myelinated axons were statistically larger in pyramid than in control (*p* = 0.032).

Myelin sheath was statistically thicker in pyramid (*p* < 0.001), fixed (*p* = 0.001), and failure (*p* = 0.011) groups compared to control.

Schwann cell nuclei were statistically larger in pyramid (*p* = 0.001), fixed (*p* = 0.002), and failure (*p* = 0.024) groups compared to control.

Microtubules and neurofilaments were greater in pyramid than in control (*p* = 0.034 and *p* = 0.004, respectively).

### Cytoflurescence

#### Plantaris endplate morphology

The statistical results of the analysis of the morphology of the postsynaptic component of the plantaris muscle indicate that no training protocol significantly altered any analyzed parameter. Data are presented in Table 2.

**TABLE 2:** Morphometry of the plantaris postsynaptic plate of the Wistar rats after 8 weeks of ladder-based resistance training of the sedentary control (control), predetermined number of climbs and progressive submaximal intensity (fixed), high-intensity pyramidal system with a predetermined number of climbs (pyramid) and high-intensity pyramidal system to failure (failure) groups.

Variables	Control	Fixed	Pyramid	Failure
*Non-stained receptors*				
*Total perimeter (µm)*	97.28 ± 8.4	106.27 ± 11.29	112.16 ± 16.56	104.81 ± 11.72
*Total area (µm* ^*2*^)	438.42 ± 45.91	453.9 ± 45.24	488.81 ± 42.65	438.34 ± 51.54
*Stained receptors*				
*Perimeter (µm)*	231.96 ± 32.88	246.64 ± 40.67	270.31 ± 39.05	242.64 ± 41.71
*Area (µm* ^*2*^)	161.56 ± 24.29	167.97 ± 20.09	171.3 ± 23.69	157.97 ± 25.69
*Dispersion (%)*	36.71 ± 1.87	36.94 ± 1.77	36.28 ± 2.63	36.42 ± 2.82

Values are presented as mean ± standard deviation. Dispersion = stained area/total non-stained area x 100.

#### Soleus endplate morphology

The statistical results of the analysis of the morphology of the postsynaptic component of the soleus muscle indicate that no training protocol significantly altered any analyzed parameter. Data are presented in Table 3.

**TABLE 3:** Morphometry of the soleus postsynaptic plate of the Wistar rats after 8 weeks of ladder-based resistance training of the sedentary control (control), predetermined number of climbs and progressive submaximal intensity (fixed), high-intensity pyramidal system with a predetermined number of climbs (pyramid) and high-intensity pyramidal system to failure (failure) groups.

Variables	Control	Fixed	Pyramid	Failure
*Non-stained receptors*				
*Total perimeter (µm)*	370.57 ± 43.68	375.07 ± 52.76	409.52 ± 62.48	403.85 ± 56.66
*Total area (µm* ^*2*^)	475.77 ± 60.43	511.35 ± 42.86	523.73 ± 51.51	515.46 ± 47.56
*Stained receptors*				
*Perimeter (µm)*	341.95 ± 44.01	346.89 ± 55.78	365.01 ± 47.29	353.89 ± 50.14
*Area (µm* ^*2*^)	306.48 ± 37.91	331.88 ± 39.05	332.99 ± 41.41	339.66 ± 39.23
*Dispersion (%)*	64.84 ± 7.21	65.05 ± 7.15	63.68 ± 6.13	66.03 ± 6.64

Values are presented as mean ± standard deviation. Dispersion = stained area/total non-stained area x 100.

#### Histochemistry

For plantaris, the comparison between groups showed a statistical difference for type i (f = 4.052, *p* = 0.015) and type ii (f = 15.052, *p* < 0.01) myofibers. the type i myofibers were statistically larger in the pyramid (*p* = 0.016) and fixed (*p* = 0.041) than in control. the pyramid, fixed, and failure groups for type ii myofibers had larger csa than control (*p* < 0.01).

For soleus, the comparison between groups showed a statistical difference for type I (F = 3.724, *p* = 0.021) and type II (F = 16.248, *p* < 0.01) myofibers. The type I myofibers were statistically larger in the pyramid than in control (*p* = 0.034). pyramid and fixed had larger csa for type ii myofibers than control and FAILURE (*p* < 0.05). Data are presented in Table 4.

**TABLE 4:** Morphometry of the plantaris and soleus muscle after 8 weeks of ladder-based resistance training of the sedentary control (control), predetermined number of climbs and progressive submaximal intensity (fixed), high-intensity pyramidal system with a predetermined number of climbs (pyramid) and high-intensity pyramidal system to failure (failure) groups.

Variables	Control	Fixed	Pyramid	Failure
*Plantaris Cross-sectional Area (µm* ^*2*^)				
*Type I*	1304.32 ± 197.88	1572.86 ± 199.99^a^	1611.08 ± 262.11^a^	1485.42 ± 133.92
*Type II*	1501.81 ± 204.07	1996.51 ± 237.77^a^	2085.29 ± 186.73^a^	1909.09 ± 162.49^a^
*Soleus Cross-sectional Area (µm* ^*2*^)				
*Type I*	2779.18 ± 623.76	3,323.75 ± 601.49	3,557.64 ± 578.25^a^	2873.98 ± 482.21
*Type II*	1957.36 ± 142.64	2739.08 ± 465.46^a^ ^,^ ^b^	2961.3 ± 310.74^a^ ^,^ ^b^	2264.64 ± 350.81

Values are presented as mean ± standard deviation—legend.

^a^ Statistical different than CONTROL.

^b^ Statistical different than FAILURE.

#### Training measurements

At the end of the experiment, the mean cumulative total mass was higher in the PYRAMID group (80,443.8 kg) compared to the FIXED (66,339.5 kg) and FAILURE (57,484.9 kg) groups.

The cumulative mass progression delta was statistically different between the FIXED (3,054.83 ± 1250.88 kg), PYRAMID (3,281 ± 956.91 kg), and FAILURE (1296.6 ± 814.6 kg) groups (F = 11.206, *p* < 0.001). The PYRAMID and FIXED groups showed greater mass progression delta than the FAILURE group (*p* < 0.05).

The number of climbs was statistically different between groups (F = 108.410, *p* < 0.001). The PYRAMID group climbed the ladder significantly more times than the FIXED and FAILURE groups (PYR = 7.32 ± 0.56; FIX = 5.85 ± 0.2; FAI = 4.67 ± 0.88, *p* < 0.001). The FIXED group statistically climbed the ladder more times than the FAILURE group (*p* < 0.001).

## Discussion

The present study showed interesting, intriguing, and, to some extent, unexpected results, especially in analyzing the morphology of the tibial nerve and motor endplate. Here, we show that the morphology of the neuromuscular system, when investigated by separate compartments, presented a non-uniform response. Furthermore, the three analyzed compartments (peripheral nerve, motor endplate, and muscle fibers from different skeletal muscles) showed other morphological characteristics after being submitted to three LRT protocols.

Krause Neto et al. (2022b) demonstrated that the radial and sciatic nerves presented a similar morphology after 8 weeks of LRT, even after being stressed by training protocols with different characteristics. Here, we observed that different LRT protocols elicited a heterogeneous response in tibial nerve morphology. Carbone et al. (2017) subjected rodents to training protocols with varying intensities of training despite the number of climbs equalizing the training volume. At the end of the experiment, the authors verified that only the group with greater intensity presented significant changes in the morphology of the radial nerve. This fact was corroborated by Krause Neto et al. (2021), whose study showed that the progression of the total mass accumulated from high-intensity training could influence the morphological adjustments of different peripheral nerves.

In the present study, we demonstrated that the morphology of the tibial nerve of the group submitted to the protocol with a greater total volume of accumulated mass had larger myelinated fibers and axons, in addition to greater thickness in the myelin sheath. Our results are corroborated by Krause Neto et al. (2021). The authors demonstrated that an LRT protocol with high volume, high intensity, and progression of accumulated training mass stimulated morphological changes in different peripheral nerves. In addition, Carbone et al. (2017) demonstrated that more significant volumes of accumulated mass induced by external load progression from higher training intensities represent a sufficient stimulus to cause ultrastructural changes. Despite this, Krause Neto et al. (2022b) demonstrated that protocols with different mass accumulations but with increasing intensities could significantly affect peripheral nerves in rodents. Therefore, it is also possible to speculate that the progression of the total training mass could be significantly associated with the accumulation of stress coming from the training intensity and not necessarily from the training volume, such as the climbing number. On the other hand, Ilha et al. (2008) indicated that nerves in traumatized conditions may be sensitive to training stimuli with lower magnitudes of intensity. This demonstrates that the adaptive response can vary between healthy nerves and pathological conditions.

Here, we demonstrate that the mean diameter of the SC nuclei was larger in the training groups compared to the control group, regardless of the type of protocol performed. Physiologically, SC produces myelin, increasing the axonal membrane’s insulation effect and the action potential’s propagation speed in myelinated axons. This morphological response may be associated with increased axonal area, mainly because the nucleus occupies 8% of the cell’s internal space (Cantwell and Nurse, 2019). Our results are corroborated by similar findings in the work of Krause Neto et al. (2021) and Krause Neto et al. (2022b).

On the other hand, we did not find any significant change in the number of SC nuclei in the present study. Our results were corroborated by Krause Neto et al. (2021) and Krause Neto et al. (2022b) but different from Carbone et al. (2017). Carbone et al. (2017) demonstrated that higher intensities of LRT (75%b.m) positively influenced the mean number of SC at the nucleus level. However, Krause Neto et al. (2021) and Krause Neto et al. (2022b) suggested that LRT-induced stress may not stimulate SC hyperplasia. These divergent results may not be directly associated with variables such as intensity or climbing volume but with the training stimulus’s weekly frequency. For example, Carbone et al. (2017) submitted the rodents to five weekly and continuous training sessions, while in other studies, the rodents trained only three times a week and on alternate days. Therefore, it is possible to suggest that a higher training frequency may cause different adjustments in the number of CS at the nucleus level.

Internally, a complex network of structures with different arrangements forms the axonal cytoskeleton, whose function is to ensure the stability of the nerve fiber. Here, only the PYRAMID group demonstrated other measures of axonal diameter, neurofilaments, and microtubules. Previous studies showed that neurofilaments determine the size of the axon, and microtubules guarantee structural rigidity (Hoffman et al., 1985; Hoffman et al., 1987; Koppers and Farías, 2021). According to Hoffman et al. (1984), there is a direct relationship between the size of axons and the content of neurofilaments. According to Matamoros et al. (2019), the increase in microtubules is essential for remodeling post-trauma axonal fibers. However, the results presented here differ from others. For example, Krause Neto et al. (2021), Krause Neto et al. (2022b), and Cabone et al. (2017) demonstrated that different training protocols induced increases in neurofilament and microtubule axons. However, none of these studies investigated such outcomes in the tibial nerve. Therefore, different peripheral nerves may present different morphological responses when submitted to different training protocols.

The NMJ region is susceptible to stimuli or their absence (Wilson and Deschenes, 2005; Krause Neto et al., 2015; Deschenes et al., 2021; Deschenes et al., 2022). However, we demonstrated that none of the LRT protocols could elicit morphological changes in the motor endplate of any of the evaluated muscles. In the original study by Deschenes et al. (2000), there was a significant difference in the perimeter (15%), area (16%), and dispersion of ACh receptors within the soleus muscle endplate region compared to the control group after 7 weeks of training. Furthermore, according to the authors, this effect was not attributed to muscle fiber hypertrophy. On the other hand, Deschenes et al. (2015) revealed that LRT-induced remodeling had been found only in the motor endplate of type II muscle fibers of the soleus muscle, without any significant change in any compartment of the NMJ in the plantaris muscle after 6 weeks of training. Indeed, the effects of LRT on postsynaptic adjustments in the NMJ are differently reported in the literature. However, it might be because of different approaches applied in the studies, such as NMJ cytofluorescence methodology and training duration. In the study of treadmill running training, Deschenes et al. (2020) did not observe changes in motor endplate after 8 weeks. However, he pointed out that prolonged exercise training (over 10 weeks) could induce morphological changes based on the exercise intensity. Therefore, it seems that the duration of the training might be an important issue in assessing postsynaptic effects. Also, according to Deschenes et al. (2020) and Deschenes et al. (2023), it is more likely that changes in the structure and size of the NMJ are associated with changes in the active zones and cellular and subcellular components of the NMJ. Therefore, we suggest further studies be conducted to assess whether LRT’s longer duration can induce changes in these parameters.

Further, Krause Neto et al. (2022a) defined muscle fiber hypertrophy in rodents as significantly correlated with total volume and delta of cumulative mass progression. Theoretically, the groups that made the most progress in the external training load are the ones that most hypertrophied their muscle fibers. Corroborating this evidence, we found that both skeletal muscles presented higher values of fCSA, as measured from the groups that showed the highest delta progression of the external training load to the average number of climbs per session. In 2017, Tibana et al. (2017) demonstrated that rodents subjected to a higher volume of climbing per training session had higher fCSA measurements at the end of the training period.

Furthermore, Luciano et al. (2017) suggested that more significant amounts of accumulated mass would be required to stimulate muscle hypertrophy, in addition to increases in mTOR phosphorylation (mammalian target of rapamycin) and regulatory enzymes of muscle hypertrophy in rodents. Finally, in humans, Damas et al. (2019) and Nóbrega et al. (2023) demonstrated that applying the progressive overload principle is fundamental for muscular hypertrophy. The sum of this evidence strengthens and confirms the findings of this study. In addition, other studies corroborate our findings (Mônico-Neto et al., 2015; Padilha et al., 2019).

On the other hand, the FAILURE group did not show fCSA values different from the CONTROL group, except for type II fibers of the plantaris muscle. The absence of significant change can be attributed to the smaller, and possibly insufficient, stimuli caused by the lower volume of climbing and progression of the absolute external training load. This result indicates a probable reason for explaining the initial effects that Hornberger and Farrar (2004) obtained. Corroborating this, Padilha et al. (2017) did not demonstrate differences in measured PRE *versus* POST of fCSA rats submitted to the “failure” protocol. However, Prestes et al. (2012) showed significant muscle hypertrophy in rats subjected to “exhaustion.” Also, this divergence in results between studies may be associated with the lack of monitoring of external load progression in many studies with rodents. Therefore, our experiment confirms that the progression of external load influences rodents’ muscular hypertrophy.

## Conclusion

The present study’s findings allow us to conclude that the LRT with greater volume and progression of accumulated mass elicits more significant changes in the ultrastructure of the tibial nerve and muscle hypertrophy of healthy rodents. In addition, protocols with moderate intensity and adequate progression of accumulated mass also induce substantial changes in the thickness of the myelin sheath and mean diameter of the SC nucleus, in addition to significant muscle hypertrophy. However, training protocols with higher levels of exhaustion may impair muscle gains and induce lower morphological responses in the tibial nerve.

Finally, no training protocol induced a significant morphological change in the motor endplate.

## Data Availability

The raw data supporting the conclusion of this article will be made available by the authors, without undue reservation.
